# Genetic Characterization of *Orientia tsutsugamushi,* Bhutan, 2015

**DOI:** 10.3201/eid3109.241763

**Published:** 2025-09

**Authors:** Tshokey Tshokey, John Stenos, Mythili Tadepalli, Chelsea Nguyen, Stephen R. Graves

**Affiliations:** Jigme Dorji Wangchuck National Referral Hospital, Thimphu, Bhutan (T. Tshokey); Flinders University, Adelaide, South Australia, Australia (T. Tshokey); Australian Rickettsial Reference Laboratory, Geelong, Victoria, Australia (T. Tshokey, J. Stenos, M. Tadepalli, C. Nguyen, S.R. Graves)

**Keywords:** Orientia tsutsugamushi, bacteria, scrub typhus, vector-borne infections, Bhutan, molecular characterization, 56 kDa gene, zoonoses

## Abstract

We performed molecular characterization of *Orientia tsutsugamushi* on DNA sequences from 5 patients from Bhutan with scrub typhus. In the 56 kDa gene, all isolates aligned with those from other Asia countries, including Nepal, India, Thailand, and Taiwan. High serum IgM titers correlated with PCR positivity in acutely ill patients.

*Orientia tsutsugamushi* is an intracellular bacterium that causes an acute febrile illness called scrub typhus. It is transmitted through the bite of infected trombiculid larva mites (chiggers). Globally, scrub typhus is a huge public health burden, mainly in the Asian tropics; overall seroprevalence is ≈25% and is higher in male than female patients (*1*). The detection of scrub typhus in the Middle East and South America is evidence that scrub typhus may be endemic beyond the traditionally described tsutsugamushi triangle in the Asia Pacific region (*1*,*2*). 

In Bhutan, scrub typhus is increasingly reported as a significant public health problem; estimated annual incidence is 62 cases/100,000 population (*3*). In 2015, ≈7% of hospitalized patients with acute febrile illnesses had scrub typhus, and a seroprevalence of ≈23% was reported in the general population (*4*). Although scrub typhus is a huge public health problem in Bhutan, data are limited to a few outbreak reports and seroepidemiologic studies with no information on genetic diversity of *O. tsutsugamushi*. This study describes the molecular characteristics of 5 *O. tsutsugamushi* sequences from Bhutan. The Bhutan Research Ethics Board of Health reviewed and approved the study. Patients provided written consent before participation.

## The Study

We used 5 real-time quantitative PCR (qPCR) positive samples and their corresponding serology results for this study. In a previous study that used the same samples (*4*), blood samples were collected from a population of acute febrile patients visiting different hospitals in Bhutan and shipped to the Australian Rickettsial Reference Laboratory (ARRL) for analysis. The samples were tested for *O. tsutsugamushi*, the causal agent of scrub typhus, by qPCR and serology. For qPCR, DNA was extracted from the buffy coat sample by using a HiYield DNA Mini Kit (Real Genomics, http://www.real-biotech.com) and tested with the qPCR established in the ARRL and validated previously (*5*). Antibody testing (initial screening followed by end titration) used the microimmunofluorescence assay established and used as the routine protocol in the ARRL (*6*). 

We amplified the *O. tsutsugamushi* samples that tested positive by qPCR with conventional 56 kDa PCR as described previously (*7*), with slight modification. Macrogen Inc. (Seoul, South Korea; https://dna.macrogen.com) sequenced the amplified DNA products (Figure). We submitted the 5 sequences to GenBank under accession numbers PQ206269, PQ206270, PQ206271, PQ206272, and PQ206273 for samples numbered Bhutan1, Bhutan3, Bhutan5, Bhutan6, and Bhutan7, respectively. In their corresponding antibody test by microimmunofluorescence assay, all 5 qPCR-positive samples had very high titers for *O. tsutsugamushi* IgM, IgG, or both, indicating acute illness (Table). 

**Figure:**
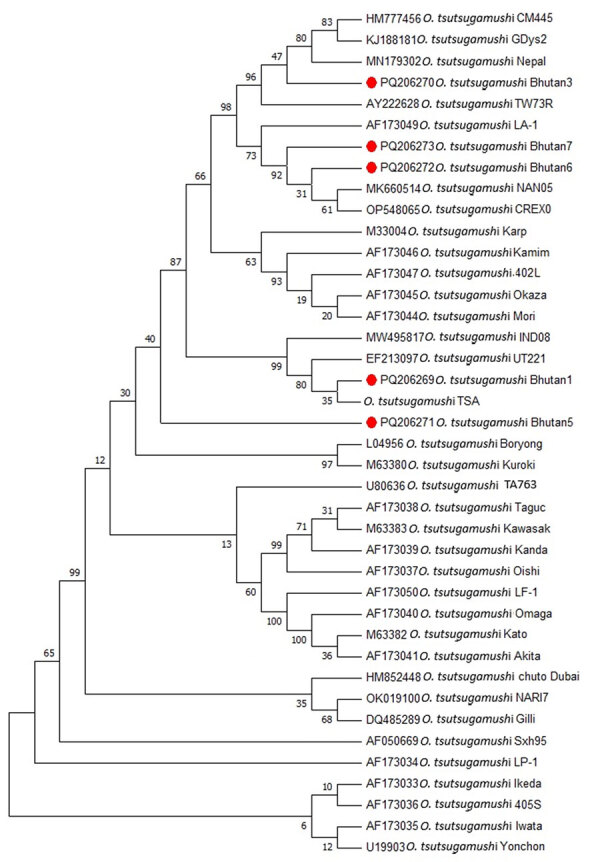
*Orientia tsutsugamushi* 56 kDa gene bootstrap consensus phylogenetic tree from genetic characterization study, Bhutan. Red dots indicate the 5 sequences characterized in this study. The tree was inferred using the neighbor-joining method. The percentages of replicate trees in which the associated taxa clustered together in the bootstrap test (1,000 replicates) are shown next to the branches. GenBank accession numbers are shown.

**Table:** Antibody test results of microimmunofluorescence assay screening and end titer for scrub typhus in study of Orientia tsutsugamushi, Bhutan, 2015*

Sample no.	Positive threshold	*Orientia tsutsugamushi* serotype test result								*Orientia chuto*	
		Gilliam			Karp			Kato			
		IgG	IgM		IgG	IgM		IgG	IgM	IgG	IgM
Bhutan1	>1:128	1:256	1:2,048		<1:128	1:4,096		<1:128	1:4,096	<1:128	<1:128
Bhutan3	>1:128	<1:128	1:2,048		<1:128	1:4,096		<1:128	1:4,096	<1:128	<1:128
Bhutan5	>1:128	<1:128	1:1,024		<1:128	1:1,024		<1:128	1:1,024	<1:128	<1:128
Bhutan6	>1:128	1:1,024	1:1,024		1:1024	1:1,024		1:1,024	1:1,024	<1:128	<1:128
Bhutan7	>1:128	1:256	1:512		1:512	1:512		1:256	1:256	<1:128	<1:128

## Conclusions

*O. tsutsugamushi* isolates from Bhutan appeared to be located in 2 main clusters in the phylogenetic tree (Figure) but are closely related. Samples Bhutan1, Bhutan3, Bhutan6, and Bhutan7 belonged to 1 cluster, and Bhutan 5 appeared to form a separate cluster. Bhutan1 was similar to an isolate *O. tsutsugamushi* karp strain UT221 from northeastern Thailand (*8*). Isolates Bhutan6 and Bhutan7 were in the same phylogenetic tree as that of *O. tsutsugamushi* CREX0 found in the Maesot and Chiangrai areas of northwestern Thailand (*9*) and in China, Japan, and South Korea (*10*). Isolate Bhutan3 was at the same level as isolates from Nepal (*11*) and Taiwan (*12*) in the phylogenetic tree. Bhutan5, which appeared to be in a different cluster from the rest of the isolates, was related to isolates from the Gorakhpur area in Uttar Pradesh, India, which is geographically nearer to Nepal and the Himalayas (*13*). Overall, all 5 isolates from Bhutan align with the Asia cluster of *O. tsutsugamushi*, as expected. None of the samples was related to *O. chuto*, which, as of July 2025, had only been identified in the Middle East (*14*) and Africa (*15*). The high serum IgM titers in the 5 patients correlated well with their qPCR positivity. That finding suggests that in acute scrub typhus infection, serologic tests that detect IgM and qPCR might be useful tools for early diagnosis, which would prompt early initiation of appropriate treatment to prevent complications. We detected *O. tsutsugamushi* antibodies against Karp, Gilliam, and Kato serotypes but no *O. chuto* antibodies, indicating that *O. chuto* is not circulating in Bhutan at the time of this study.

The main limitation of this study was that 5 DNA samples were available for sequencing and only the 56 KDa gene was sequenced. That gene is the one most commonly used for phylogenetic analysis because it contains the most polymorphisms. Thus, this molecular report from Bhutan presents a preliminary genetic characterization of *O. tsutsugamushi*. Studies that include more samples and sequencing of additional gene targets will confirm characterizations.

In summary, we characterized 5 *O. tsutsugamushi* sequences from patients in Bhutan and found that they mostly align with isolates from other countries in Asia. Serologic testing for IgM and qPCR testing can provide early diagnosis of acute scrub typhus infection and timely initiation of treatment to prevent complications. 

## References

[1] Dasgupta S, Asish PR, Rachel G, Bagepally BS, Chethrapilly Purushothaman GK. Global seroprevalence of scrub typhus: a systematic review and meta-analysis. Sci Rep. 2024;14:10895. 10.1038/s41598-024-61555-9 38740885 PMC11091130

[2] Weitzel T, Dittrich S, López J, Phuklia W, Martinez-Valdebenito C, Velásquez K, et al. Endemic scrub typhus in South America. N Engl J Med. 2016;375:954–61. 10.1056/NEJMoa1603657 27602667

[3] Dorji K, Phuentshok Y, Zangpo T, Dorjee S, Dorjee C, Jolly P, et al. Clinical and epidemiological patterns of scrub typhus, an emerging disease in Bhutan. Trop Med Infect Dis. 2019;4:56. 10.3390/tropicalmed4020056 30934849 PMC6631561

[4] Tshokey T, Stenos J, Durrheim DN, Eastwood K, Nguyen C, Vincent G, et al. Rickettsial infections and Q fever amongst febrile patients in Bhutan. Trop Med Infect Dis. 2018;3:12. 10.3390/tropicalmed3010012 30274410 PMC6136613

[5] Stenos J, Graves S, Izzard L. Rickettsia. In: Schuller M, Sloots TP, James GS, Halliday CL, Carter IWJ, editors. PCR for clinical microbiology: an Australian and international perspective. Dordrecht (the Netherlands): Springer; 2010. p. 197–9.

[6] Graves SR, Dwyer BW, McColl D, McDade JE. Flinders Island spotted fever: a newly recognised endemic focus of tick typhus in Bass Strait. Part 2. Serological investigations. Med J Aust. 1991;154:99–104. 10.5694/j.1326-5377.1991.tb120994.x 1898756

[7] Unsworth NB, Stenos J, Faa AG, Graves SR. Three rickettsioses, Darnley Island, Australia. Emerg Infect Dis. 2007;13:1105–7. 10.3201/eid1307.050088 18214193 PMC2878210

[8] Paris DH, Aukkanit N, Jenjaroen K, Blacksell SD, Day NPJ. A highly sensitive quantitative real-time PCR assay based on the groEL gene of contemporary Thai strains of *Orientia tsutsugamushi.* Clin Microbiol Infect. 2009;15:488–95. 10.1111/j.1469-0691.2008.02671.x 19416296 PMC3429864

[9] Rungrojn A, Batty EM, Perrone C, Abdad MY, Wangrangsimakul T, Brummaier T, et al. Molecular diagnosis and genotyping of *Orientia tsutsugamushi* in Maesot and Chiangrai, Thailand. Front Trop Dis. 2023;4:1146138. 10.3389/fitd.2023.1146138 39376595 PMC7616666

[10] Enatsu T, Urakami H, Tamura A. Phylogenetic analysis of *Orientia tsutsugamushi* strains based on the sequence homologies of 56-kDa type-specific antigen genes. FEMS Microbiol Lett. 1999;180:163–9. 10.1111/j.1574-6968.1999.tb08791.x 10556707

[11] Gautam R, Parajuli K, Tadepalli M, Graves S, Stenos J, Sherchand JB. Scrub typhus and molecular characterization of *Orientia tsutsugamushi* from central Nepal. Pathogens. 2021;10:422. 10.3390/pathogens10040422 33916224 PMC8066985

[12] Qiang Y, Tamura A, Urakami H, Makisaka Y, Koyama S, Fukuhara M, et al. Phylogenetic characterization of *Orientia tsutsugamushi* isolated in Taiwan according to the sequence homologies of 56-kDa type-specific antigen genes. Microbiol Immunol. 2003;47:577–83. 10.1111/j.1348-0421.2003.tb03420.x 14524618

[13] Nanaware N, Desai D, Banerjee A, Zaman K, Mittal M, Mittal M, et al. Genotypic characterization of *Orientia tsutsugamushi* isolated from acute encephalitis syndrome and acute febrile illness cases in the Gorakhpur Area, Uttar Pradesh, India. Front Microbiol. 2022;13:910757. 10.3389/fmicb.2022.910757 35865917 PMC9294505

[14] Izzard L, Fuller A, Blacksell SD, Paris DH, Richards AL, Aukkanit N, et al. Isolation of a novel *Orientia* species (*O. chuto* sp. nov.) from a patient infected in Dubai. J Clin Microbiol. 2010;48:4404–9. 10.1128/JCM.01526-10 20926708 PMC3008486

[15] Masakhwe C, Linsuwanon P, Kimita G, Mutai B, Leepitakrat S, Yalwala S, et al. Identification and characterization of *Orientia chuto* in trombiculid chigger mites collected from wild rodents in Kenya. J Clin Microbiol. 2018;56:e01124–18. 10.1128/JCM.01124-18 30282787 PMC6258837

